# Considerations for Improving Metabolism Predictions for *In Vitro* to *In Vivo* Extrapolation

**DOI:** 10.3389/ftox.2022.894569

**Published:** 2022-04-29

**Authors:** Marjory Moreau, Pankajini Mallick, Marci Smeltz, Saad Haider, Chantel I. Nicolas, Salil N. Pendse, Jeremy A. Leonard, Matthew W. Linakis, Patrick D. McMullen, Rebecca A. Clewell, Harvey J. Clewell, Miyoung Yoon

**Affiliations:** ^1^ ScitoVation, LLC, Durham, NC, United States; ^2^ Oak Ridge Institute for Science and Education, Oak Ridge, TN, United States; ^3^ Ramboll US Corp., Research Triangle Park, NC, United States; ^4^ 21st Century Tox Consulting, Chapel Hill, NC, United States

**Keywords:** IVIVE, *in vitro*, metabolism, QSAR, HT-IVIVE, risk assessment

## Abstract

High-throughput (HT) *in vitro* to *in vivo* extrapolation (IVIVE) is an integral component in new approach method (NAM)-based risk assessment paradigms, for rapidly translating *in vitro* toxicity assay results into the context of *in vivo* exposure. When coupled with rapid exposure predictions, HT-IVIVE supports the use of HT *in vitro* assays for risk-based chemical prioritization. However, the reliability of prioritization based on HT bioactivity data and HT-IVIVE can be limited as the domain of applicability of current HT-IVIVE is generally restricted to intrinsic clearance measured primarily in pharmaceutical compounds. Further, current approaches only consider parent chemical toxicity. These limitations occur because current state-of-the-art HT prediction tools for clearance and metabolite kinetics do not provide reliable data to support HT-IVIVE. This paper discusses current challenges in implementation of IVIVE for prioritization and risk assessment and recommends a path forward for addressing the most pressing needs and expanding the utility of IVIVE.

## Introduction

Widespread implementation of risk assessment strategies based on *in vitro* methods requires fundamental changes in how safety evaluations and decisions are made, along with well-defined frameworks for the use of *in vitro* experiments coupled with high throughput (HT) computational tools (new approach methods; NAMs) to meet the needs of the ever-evolving regulatory, scientific, and legislative landscapes. Rapid progress in development of new *in silico* and *in vitro* methods is facilitating the movement away from animal studies and should help to increase confidence in chemical safety decisions based on new approach methodologies (NAMs). Recent case studies have demonstrated that *in silico* tools, such as the threshold for toxicological concern (TTC), and HT *in vitro* assays coupled with HT *in vitro* to *in vivo* extrapolation (HT-IVIVE) generally provide conservative estimates for chemical points of departure (PoD), and therefore offer a viable alternative to the use of traditional approaches for prioritizing chemicals based on potential risk (Patlewicz et al., 2018).

HT-IVIVE, a critical tool for translating *in vitro* bioactivity into estimated human *in vivo* exposures, uses values for parent chemical loss via metabolism, renal clearance, plasma binding, and absorption to predict external exposures that would give rise to steady state parent chemical plasma concentrations equivalent to active concentrations in the *in vitro* test medium (Rotroff et al., 2010; Wetmore et al., 2013; Sipes et al., 2017). To do this, *in vitro* metabolism data is necessary, together with pharmacokinetic (PK) modeling, in a process referred to as reverse dosimetry (Clewell et al., 2008). While this process is essential to translating *in vitro* bioactivity measurements to human *in vivo* exposures, several limitations in the implementation of HT-IVIVE to the broader chemical Universe exist. Due to challenges with analytical chemistry, cell culture and financial limitations, metabolism data is typically collected in short-term incubations with microsomes or primary hepatocytes with readouts for parent chemical loss. Thus, in the majority of HT uses, IVIVE considers the parent chemical as the only potentially bioactive moiety. Further, expansion of HT-IVIVE to chemicals beyond the 301 chemicals measured in Wetmore et al. (2015) has been limited by the cost and time required to develop necessary analytical chemistry methods. Here, our main goal was to evaluate approaches for incorporating metabolism information into HT-IVIVE approaches, discuss their domains of applicability and recommend research strategies for rapid improvement of current HT-IVIVE capabilities.

### Evaluation of Current Tools for Clearance Predictions

#### Published *in vitro* Metabolism Data

For cost and time efficiency, it is common to examine literature data as a source for metabolism characterization. Most published metabolic clearance data are measured using subcellular fractions, such as microsomes and cytosol, or primary cell monoculture systems. These systems are typically derived from the liver of the target species, though clearance from other tissues such as intestine, lung, and kidney may be reported when extrahepatic metabolism is known to be important for a particular chemical. Incubation with subcellular fractions or primary hepatocytes are generally performed over a few hours, as loss of enzyme activity occurs quickly *in vitro* (Nussler et al., 2001; Wilk-Zasadna et al., 2015; Cassim et al., 2017).

The greatest challenge in using existing literature data for parameterizing IVIVE models resides is the fact that *in vivo* metabolism is an integrated process involving several competing and/or interacting reactions, which may not be completely captured by the more simplified model systems that are typically used. Thus, while microsomal fractions are used often in the published literature, the domain of applicability for these methods is limited to a subset of the chemical Universe that relies on phase I cytochrome-P450-mediated oxidative reactions, carboxylesterases and epoxide hydrolases achieved with traditional microsomal incubations. While phase II metabolism via glucuronide conjugation may also be captured by microsomal preparations, other enzymes, such as the soluble phase II enzymes (sulfotransferases, glutathione s-transferases, etc.,) are neglected (Taylor and Triggle, 2007; Decker et al., 2009). While isolated cytosolic fractions may be used to evaluate these processes, this approach is far less common than the use of microsomes. Additionally, since specific cofactors must be added to facilitate some of the cytosolic enzymes, it can be difficult to find published cytosolic enzyme data for all but the most thoroughly studied chemicals. Liver S9 fraction or homogenate can also be used as they contain the major phase I and II enzymes. However their preparation is laborious (Pelkonen et al., 2009). Human liver slices have the advantage of having a preserved basic hepatic architecture, with an intact cell system and the major phase I and II enzymes. The only drawbacks are the difficulty to obtain the human liver slices and the specialized skills needed (de Graaf et al., 2010).

For the reasons described above, freshly isolated primary hepatocytes are regarded as the most relevant experimental system to study both hepatic metabolism and metabolite-mediated effects of chemicals and pharmaceuticals. Primary hepatocytes express most of the proteins found in the human liver, including those involved in metabolism, membrane transport, and receptor-mediated processes (Li et al., 1995; Vildhede et al., 2015; Yang et al., 2015). Primary hepatocytes in suspension are the gold standard for incubation periods up to 4 h but their viability is time limited (Smith et al., 2012). Plated primary hepatocytes are also the gold standard but for incubation periods longer than 4 h (Smith et al., 2012; Ma et al., 2017). However, a major drawback with plated primary hepatocytes is the rapid change in phenotype observed in culture; enzyme activity changes dramatically in the first hours after isolation (for fresh hepatocytes) or thawing (for cryopreserved hepatocytes). In most hepatocytes, the enzymatic activity decreases by approximatively 50% in the first 5–6 h, with a 95% reduction in enzyme activity within 30 h for most preparations (Nussler et al., 2001; Cassim et al., 2017). To overcome the short-life span of these preparations, several techniques have been developed to stabilize hepatic phenotype over longer periods (Vinci et al., 2010a; Vinci et al., 2010b; Ballard et al., 2016). However, these methods have not been routinely used for metabolism studies. Thus, current published metabolism data are subject to the limitations of short-term *in vitro* assays, including an inability to measure clearance of slowly metabolized compounds, which will be discussed in more detail in later sections.

Another common issue encountered when using literature-derived results for subcellular fractions or isolated hepatocytes is the effect of incubation conditions, such as protein content and substrate concentrations, on estimated intrinsic clearance (CL_int_). Estimates can vary widely for the same compound due to differences in the experimental protocol. To demonstrate this variability, we compared the published human clearance data on the conversion of the well-studied compound, bisphenol A (BPA), to its glucuronide metabolite (Table 1). All of these studies used different incubation conditions, and the resulting intrinsic clearance rates differ by more than an order of magnitude. It should be noted that different *in vitro* conditions could lead to different *in vitro* binding, which may be a significant source of variability, particularly depending on how it is (or is not) addressed (Proença et al., 2021).

**TABLE 1:** Metabolism kinetics of BPA found in the literature.

References	*V*_max_	*K*_M_ (µM)	CL_int_invitro_	CL_int_invivo_ (L/h)^a^	Metabolic system
Kuester and Sipes (2007)	438 pmol/min/10^6^ cells	9	56 μl/min/10^6^ cells	590^b^	Cryopreserved hepatocytes
Elsby et al. (2001)	5.9 nmol/min/mg protein	77.5		292^c^	Pooled liver microsomes
Kurebayashi et al. (2010)	10.6 ml/h/10^6^ cells	5.3	2 ml/h/10^6^ cells	351^b^	Cryopreserved hepatocytes
Trdan Lušin et al. (2012)	8.5 nmol/min/mg protein	8.9	0.95 ml/min/mg protein	3637^c^	Liver microsomes
Mazur et al. (2010)	2077 pmol/min/mg protein	3.6	649 μl/min/mg protein	2486^c^	Liver microsomes
Rotroff et al. (2010)			25.04 μl/min/10^6^ cells	264^b^	Hepatocytes

a Calculated *V*_max_ = maximal velocity of metabolic clearance; *K*_M_, Michaelis-Menten parameter (half-maximal metabolism concentration)

b CL_int_invivo_ = CL_int_invitro_ × HPGL (hepatocytes per gram liver: 110 million cells/g liver) × Volume liver (1,596 g) × 60 min/h.

c CL_int_invivo_ = CL_int_invitro_ × MPPGL (mg of microsome protein/g liver: 40) × Volume liver (1,596 g) × 60 min/h.

Thus, in identifying and using data from published studies, care must be taken to evaluate the study conditions and avoid using clearance predictions outside the bounds of the data. For example, studies that report rates of clearance using a single concentration should only be used if the chemical concentration was below the level of enzyme saturation. Another challenge is that studies will often provide results with insufficient detail to reproduce the calculations. These types of difficulties are quite common when looking at *in vitro* metabolic literature and can pose a significant challenge when using these results to perform HT-IVIVE.

#### Available *In Silico* Metabolism Models

A large number of *in silico* models have been developed over the years for predicting metabolism based on QSARs (Ekins and Obach 2000; Lee et al., 2007; Li et al., 2009; Lombardo et al., 2014; Sarigiannis et al., 2017; Sipes et al., 2017). These models typically take advantage of the large (but often proprietary) data sets developed during drug candidate screening, and are often focused on the development of classification models (e.g., metabolized vs. not metabolized), enzyme substrate identification (Holmer et al., 2021), metabolite identification (de Bruyn Kops et al., 2021), or prediction of metabolism by a particular class of enzymes (Mazzolori et al., 2019; Sweeney and Sterner 2022), rather than quantitative prediction of total clearance. Models developed primarily from pharmaceutical compound data share a common deficiency from the viewpoint of their application to chemicals other than drugs, in that the characteristics that make a compound suitable for use as a drug are often quite different from the characteristics of compounds of environmental and occupational interest (Leanard 2019). The properties considered important for a potential oral drug are those characteristics that will provide high bioavailability (as described in Lipinski et al., 2016) as follows:

• nonvolatile
• water soluble
• moderate to high permeability
• low lipophilicity
• not highly ionized at pH = 7.4
• low to moderate clearance
• good stability in plasma

Environmental chemicals, however, are not subject to these limitations as they are designed for many purposes other than efficient biological uptake. Moreover, while there are more than 50 CYP450 enzymes, a small subset (1A2, 2B6, 2C8, 2C9, 2C19, 2D6, 3A4/5) metabolize 90% of drugs, and important CYPs for environmental and occupational inhalation exposures (*e.g.*, 1A1, 2E1, and 2F1) are seldom considered in drug development. Thus, the utility of models based on drug data for predicting metabolic transformations for environmental chemicals is uncertain.

**FIGURE 1:**
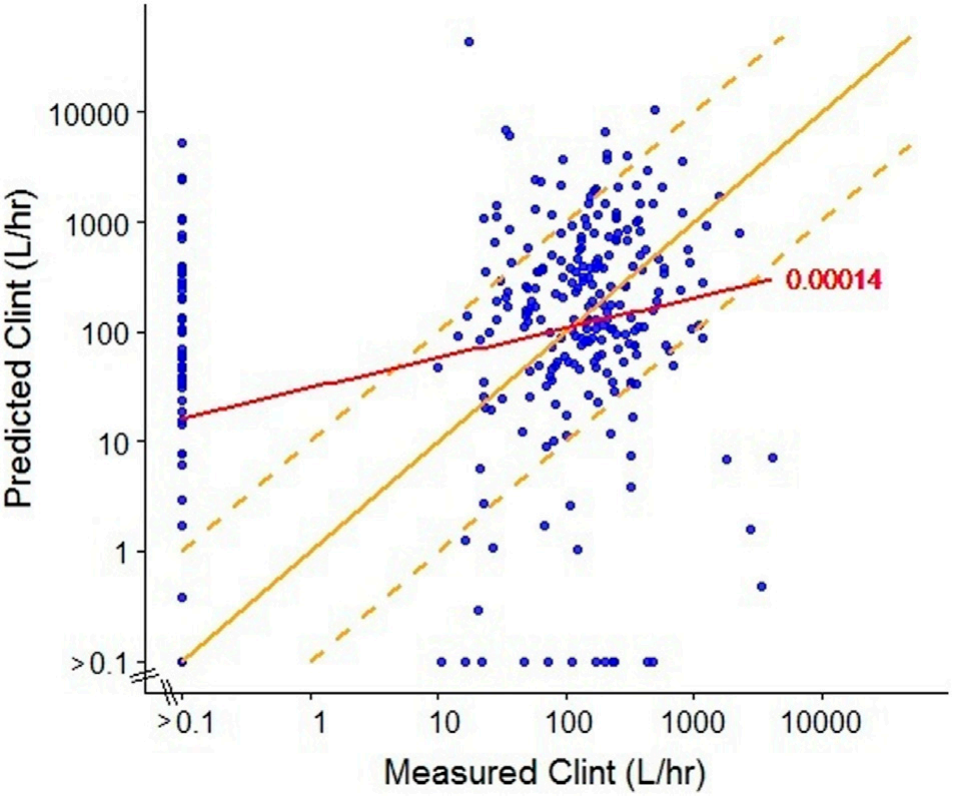
Comparison of measured (Wetmore et al., 2015) and predicted (ADMET predictor, ver7.1, SimulationsPlus) intrinsic clearance (CL_int_) for a subset of ToxCast chemicals. The red line represents the linear regression with *R* ^2^ = 0.00014. The solid yellow line represents equivalence between predicted and measured values (y = x) and the dashed yellow lines represent a 10-fold divergence between predicted and measured values (y = 10 × x; y = x/10).

We compared human *in vivo* CL_int_ estimates from one of the commonly used in silico models, ADMET predictor (Simulations Plus, ver. 7.1), which is trained using data from pharmaceuticals, for 301 ToxCast chemicals with CL_int_ estimates derived from *in vitro* hepatocyte metabolism studies (Wetmore et al., 2015) as published in Sipes et al. (2017). The relationship between experimentally measured and predicted CL_int_ values is shown in Figure 1. Overall, the *in vitro* experimental and predicted values are not well-correlated (*r* ^2^ = 0.00014), a finding that is at least partially due to the fact that the training set for the predictive tool was developed based on data from pharmaceuticals, while the chemicals from ToxCast likely have a much broader range of physicochemical properties. Additionally, inadequacies of the *in vitro* assays to which the predictions are compared may also play a role (see next section).

**FIGURE 2:**
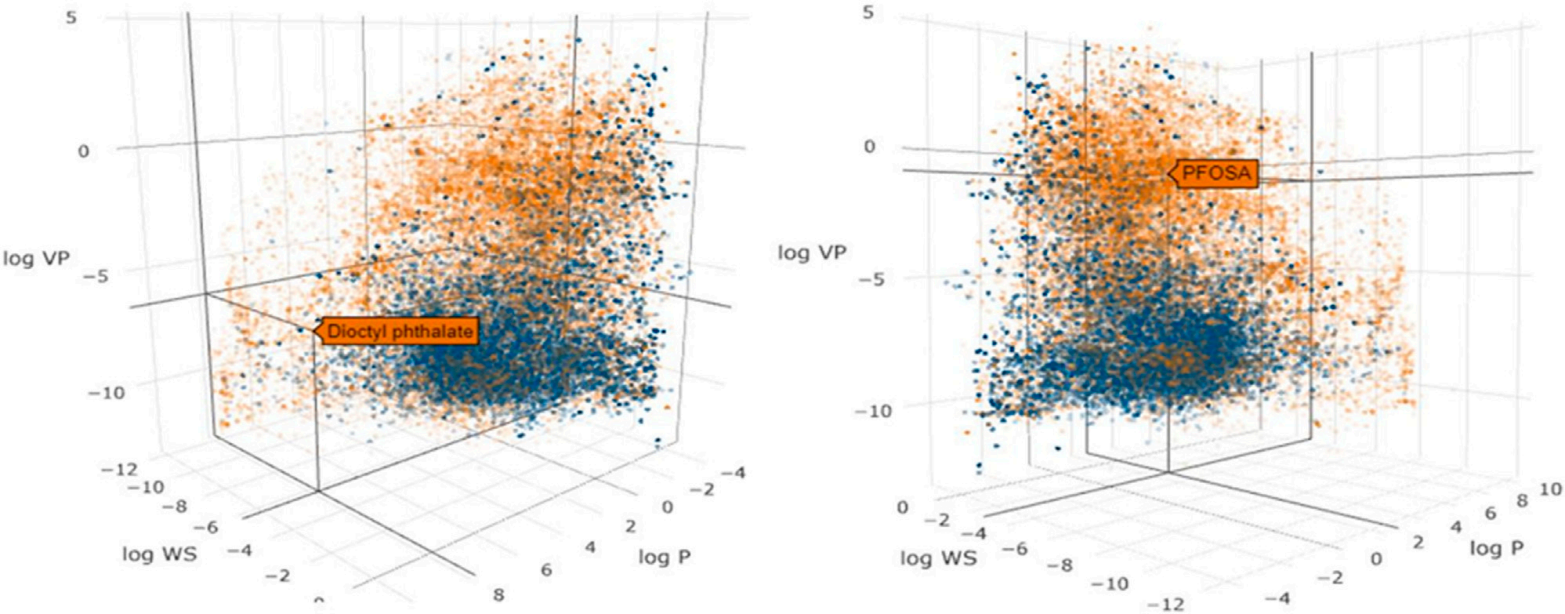
Scatter plot of the physicochemical properties of environmental chemicals (gold dots) and pharmaceuticals (blue dots) in the CERAPP database. The axes represent vapor pressure (log VP), water solubility (log WS) and lipophilicity (log P). Two different views are shown to illustrate the properties of the environmental chemicals, dioctyl phthalate (left) and PFOSA (right).

Since many of the tools used for IVIVE analysis were originally developed for pharmaceuticals, their performance against the more diverse set of chemicals encountered in the environment needs to be properly evaluated (Bell et al., 2017). To investigate the similarity in the physical-chemical properties across pharmaceutical and environmental chemicals, we used the chemical descriptor information from the Collaborative Estrogen Receptor Activity Prediction Project (CERAPP) database (Mansouri et al., 2016) to visualize the relationship between the chemical space of pharmaceutical and environmental chemicals (Figure 2). Model descriptors were physical-chemical properties from OPERA, as described in Mansouri et al. (2016): vapor pressure (logVP), water solubility (logWS), and lipophilicity (logP). Figure 2 demonstrates that pharmaceuticals only represent a subset of the chemical space that is associated, as expected, with low volatility, low lipophilicity and high water solubility.

**FIGURE 3:**
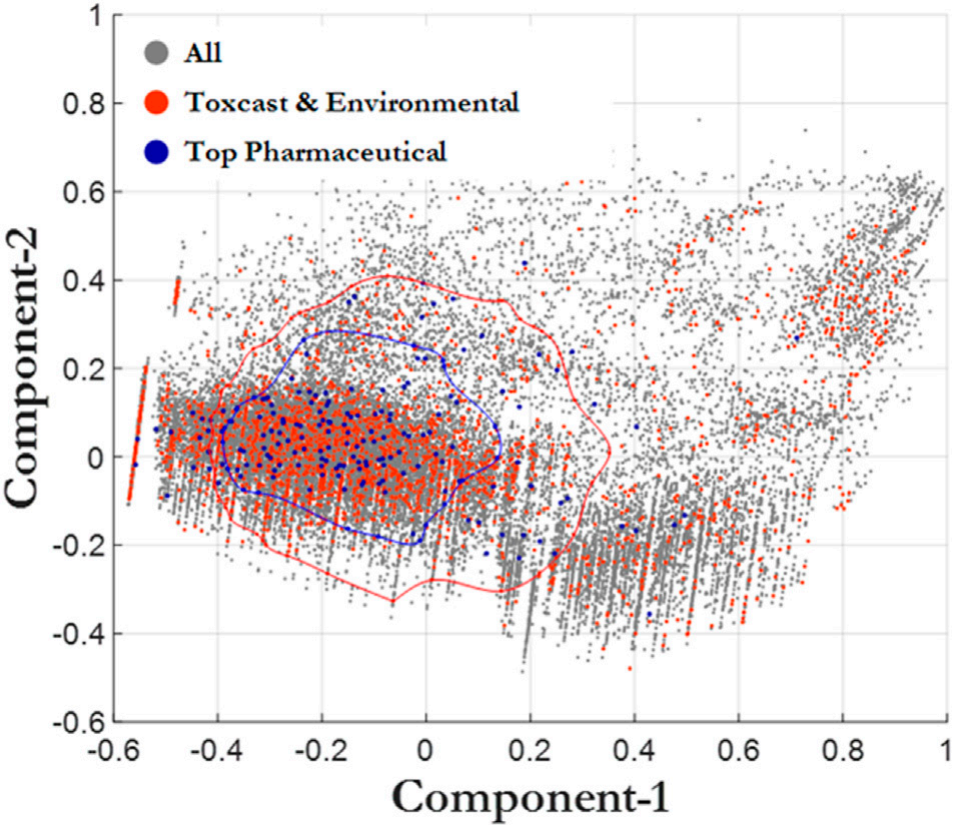
Principal component analysis. All chemicals (CERAPP, gray), environmental chemicals from ToxCast (red), and top prescribed drugs (blue). The blue bubble surrounds the Top Pharmaceutical compounds that are one standard deviation away from the mean. The red bubble surrounds the ToxCast and Environmental compounds that are one standard deviation away from the mean.

We further evaluated the chemical space of the 45,000 chemicals in the CERAPP database using principal component analysis (PCA), which is useful for determining descriptors that explain variance (Figure 3). Random Forest analysis was utilized for predicting three exposure proximity classes: near-field (NF), far-field (FF), and pharmaceutical (Rx), after which a principal component analysis was employed using the highest performing decision algorithm. A classification accuracy of 79% was achieved when simultaneously utilizing two descriptor sets: 1) CERAPP physicochemical property with Lapinski descriptors and 2) structural signatures in the form of DSSTox Toxprint chemotypes (Yang et al., 2015). The most important descriptors identified by the analysis were molecular weight and polar surface area, both of which had an importance of greater than 0.9 for the first and second components, respectively, while all other components were less than 0.3. The analysis also indicated that molecule flexibility and lipophilicity were the major factors distinguishing the pharmaceutical and environmental chemical spaces, with drug-like compounds being confined in a narrower space than environmental chemicals. Many pharmaceutical compounds contain rigid ring-type structures that limit their flexibility. This helps enhance their affinity for the target enzyme by limiting the entropic price of binding. Log *p* values for pharmaceuticals tend to be moderate: high enough to allow passage through cell membranes, while still low enough to avoid issues with bioavailability and solubility (Lipinski 2016). As environmental chemicals are not designed to meet the same criteria, they do not have these features constrained. To assess the significance of this discrepancy, we calculated the Frobenius distance (Euclidian norm of (D1-D2)) between pairs of distributions. The resulting distances (Rx-NF = 2.35, Rx-FF = 2.58, NF/FF = 0.48) support the conclusion that the Rx distribution can be seen as distinctly different from the other two, indicating that validation of an assay with pharmaceuticals may not adequately ensure the usefulness of the assay for environmental chemicals.

A number of recent efforts have attempted to address the insufficiency of pharmaceutical data for training quantitative structure activity relationship (QSAR) prediction of CL_int_ for environmental chemicals by including available data on non-pharmaceuticals, but with limited success (Pradeep et al., 2020; Dawson et al., 2021a,b; Mansouri et al., 2021). Three models, all freely available (including OPERA, which has a downloadable graphic user interface) are described herein. Pradeep et al. (2020) used a combination of read-across and QSAR to classify 524 pharmaceuticals, food-use chemicals, pesticides, and industrial chemicals into low, medium, and high CL_int_ groups. They then used a series of machine learning methods with ≥ 79 descriptors to develop predictive models for CL_int_, based on the “medium CL_int_” chemicals (n = 337). Unfortunately, the fit to the observed data was still fairly poor (test set *r* ^*2*^ = 0.14), and the model was not correlated to CL_int_ predictions from the ADMET software (*r* ^*2*^ = 0.17). Poor model performance was attributed to uncertainty and variability in the underlying *in vitro* data, as well as the possibility that important chemical descriptors had yet to be identified. Mansouri et al. (2021) developed a similar capability in the OPERA QSAR app (https://ntp.niehs.nih.gov/whatwestudy/niceatm/comptox/ct-opera/opera.html). OPERA has a chemical domain of 1,056 non-specific chemicals and utilizes QSAR, classification (cleared vs. non-cleared), and then regression on cleared chemicals to estimate CL_int_. Ultimately, Mansouri et al. (2021) noted a bimodal distribution of estimated values for CL_int_ (centered around ∼10^−6.5^ and ∼10^1.5^l/hr). The *r* ^2^ value for this model was 0.40, which still demonstrates a fair amount of room for improvement, but is much improved over the previous models. Dawson et al. (2021) built a QSAR model with 40 descriptors using both pharmaceutical and non-pharmaceutical chemicals pulled from ToxCast and ChEMBL databases. Chemicals were classified into very slow, slow, and fast/very fast metabolism, and random forest regression was performed to provide quantitative estimates of CL_int_. The overall average accuracy for the full training set was 58.7% in the training set; however, after filtering to ensure that chemicals were within the applicability domain, the accuracy when applied to ToxCast chemicals was 70.4%. Predictions were poor in the “very slow” category, but this was likely due to a severe under-representation of “slow” chemicals in the data (8% of the test set). The overall *r* ^2^ of the observed vs. predicted random forest regression was 0.52, which indicates some utility for application in early prioritization. Overall, these efforts demonstrate the importance of using relevant data model training and highlight the need for additional CL_int_ data collection in the environmental chemical space. The lack of human *in vivo* metabolism data for chemicals that are not pharmaceuticals is a limiting factor in determining the domain of applicability for *in vitro* systems. To address this deficiency, it will be necessary to conduct parallel evaluations in human and rodent tissues to extend the available *in vivo* data.

### Recommendations for Improving Intrinsic Clearance Predictions

#### Using Improved *in vitro* Systems to Collect Data to Support Expansion of Current *in Silico* Models

Two major areas for future development that were identified from our analysis of current HT-IVIVE capabilities were 1) improved *in vitro* models that allow for *in vivo* -like metabolite profiles over longer periods of time and 2) expanded domain of applicability of clearance prediction models by collecting data from the environmental chemical space. In particular, the prediction of slowly cleared environmental compounds is hindered due to the lack of experimental data. On the basis of a purely practical consideration, it is not possible to determine CL_int_ with confidence if rates of metabolism are significantly less that the life-span/duration of metabolic competence of *in vitro* hepatocytes or metabolism assay preparations. Thus, the limitations of enzyme viability in current *in vitro* models is a clear technological gap that must be addressed if predictive models are to be developed for the broader chemical Universe.

**FIGURE 4:**
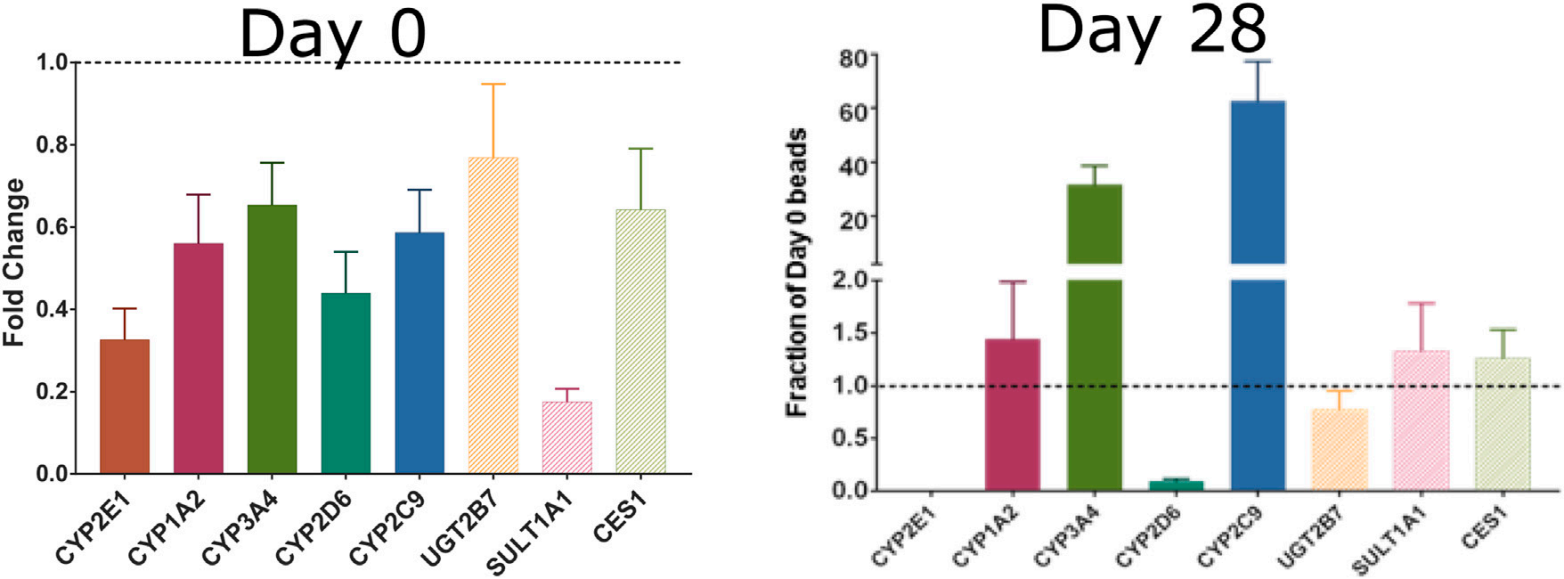
Expression of Phase I and II metabolism genes in freshly plated rat hepatocytes (left) or rat hepatocytes cultured for 28 days in alginate beads. Reproduced from Phillips et al., 2018.

The short-lived nature of most existing *in vitro* models, including the hepatocyte assay used for several HT-IVIVE studies (Rotroff et al., 2010; Wetmore et al., 2015), limits their utility for the broader Universe of compounds. Recent developments in 3D and dynamic tissue cultures, *e.g.*, bioreactors, have shown promise for improving estimates of both chemical metabolism and toxicological response (LeCluyse et al., 2012). For example, Phillips (2018) developed a 3D primary human hepatocyte cell culture system using alginate hydrogel beads with extended viability and metabolic competence that can be used for long-term primary human hepatocyte culture model with slowly metabolized chemicals. Hepatocytes are viable for more than 4 weeks in this system. Increasing the length of time that hepatocytes could be exposed to chemicals would contribute to addressing the major shortcoming of currently available *in vitro* metabolic clearance determination tools in accurately measuring slow clearance. These longer-lived *in vitro* metabolism tools would also be useful in improving coverage of Phase II metabolic processes and other clearance pathways. As an example, we measured the expression of several Phase I and Phase II metabolism genes in primary hepatocytes cultured in a 3D alginate bead system (Phillips et al., 2018). As opposed to standard suspension cultures that lose most of their RNA expression in the first day and lose viability in less than 1 week (LeCluyse et al., 2012), the hepatocytes in this 3D model showed increased expression of most of the measured genes after 4 weeks in culture (Figure 4). Such systems also hold tremendous promise for measuring low abundance or slowly formed metabolites. Bernasconi et al. (2019) showed in their study that cryopreserved PHH and cryopreserved HepaRG cells are reliable and relevant *in vitro* methods for the assessment of human CYP enzyme induction. HepaRG cell lines are metabolic competent “hepatocyte-like but they only represent one donor (Bell et al., 2017). Organoids based on primary hepatic material are also systems that have a close resemblance to *in vivo* physiological situation but there is limited information on their performance (Gough et al., 2021).

Collection of metabolism data in these improved models is nonetheless hindered by the lack of resources for generation of *in vitro* clearance data, the high cost of analytical chemistry, and the low-throughput nature of existing assays. However, we propose that the domain of applicability of current *in silico* metabolism models could be substantially improved while minimizing costs, by strategically selecting a finite set of chemicals that could be used to re-train/update current QSAR models of metabolism. Using computational approaches for defining chemical space, such as those demonstrated in the included chemical space analyses, it is possible to select specific chemicals for testing that target chemical domains of interest. Further, using non-targeted analytical chemistry techniques, we may be able to expand the throughput for metabolism studies (Sobus et al., 2018).

### *In Vitro* to *In Vivo* Extrapolation in a Risk Assessment Context

HT-IVIVE estimates of human equivalent dose using only parent chemical clearance have been successfully used to support prioritization or screening-level risk evaluations from HT *in vitro* bioactivity data (Rotroff et al., 2010; Yoon et al., 2012; Wetmore et al., 2013; Wetmore et al., 2015; Sipes et al., 2017; Casey et al., 2018; Wambaugh et al., 2018). However, when used with data from biologically relevant fit-for-purpose assays (Clewell et al., 2016; Hartman et al., 2018; Beames et al., 2020) to derive quantitative estimates of risk, more sophisticated IVIVE models (quantitative-IVIVE; Q-IVIVE) that account for issues such as slow clearance, active transport, extra-hepatic metabolism, and metabolic bioactivation are likely to be necessary (Yoon et al., 2012; 2013; 2016).

In a tiered approach to NAM-based risk assessment as described in Andersen et al. (2019), rapid computational methods that allow for increased uncertainty may be used to make rapid decisions on chemical prioritization (Level 1), and HT or organotypic *in vitro* assays may be used to make more quantitative assessments (Level 2–4). The progression between bioactivity linked risk-based evaluations at different tiers is governed by decision context and the degree of confidence required for the decision. For example, if HT methods can determine that human effect levels predicted using computational methods or *in vitro* points of departure are sufficiently large relative to likely human exposure (*i.e.*, the margin of exposure, MOE), a larger degree of uncertainty in the metabolic characterization may be tolerated in decision-making. Conversely, if an early-tier assessment predicts a small margin of exposure with higher uncertainty, successive tiered testing can be used to refine the risk estimate and gain confidence in NAM-based decisions.

In this schema, HT-IVIVE supported by *in silico* predictions of parent chemical clearance would support rapid prioritization and data collection in more organotypic metabolism models would support higher tier, quantitative assessments. However, as the domain of applicability of current data and predictive models are heavily biased toward the pharmaceutical chemical space, current efforts suggest that even as a first pass, CL_int_ predictions are in need of improvement. This situation becomes even more pressing when considering the lack of consideration of metabolite bioactivity.

### Considering Metabolite Exposure With *In Vitro* to *In Vivo* Extrapolation

**FIGURE 5:**
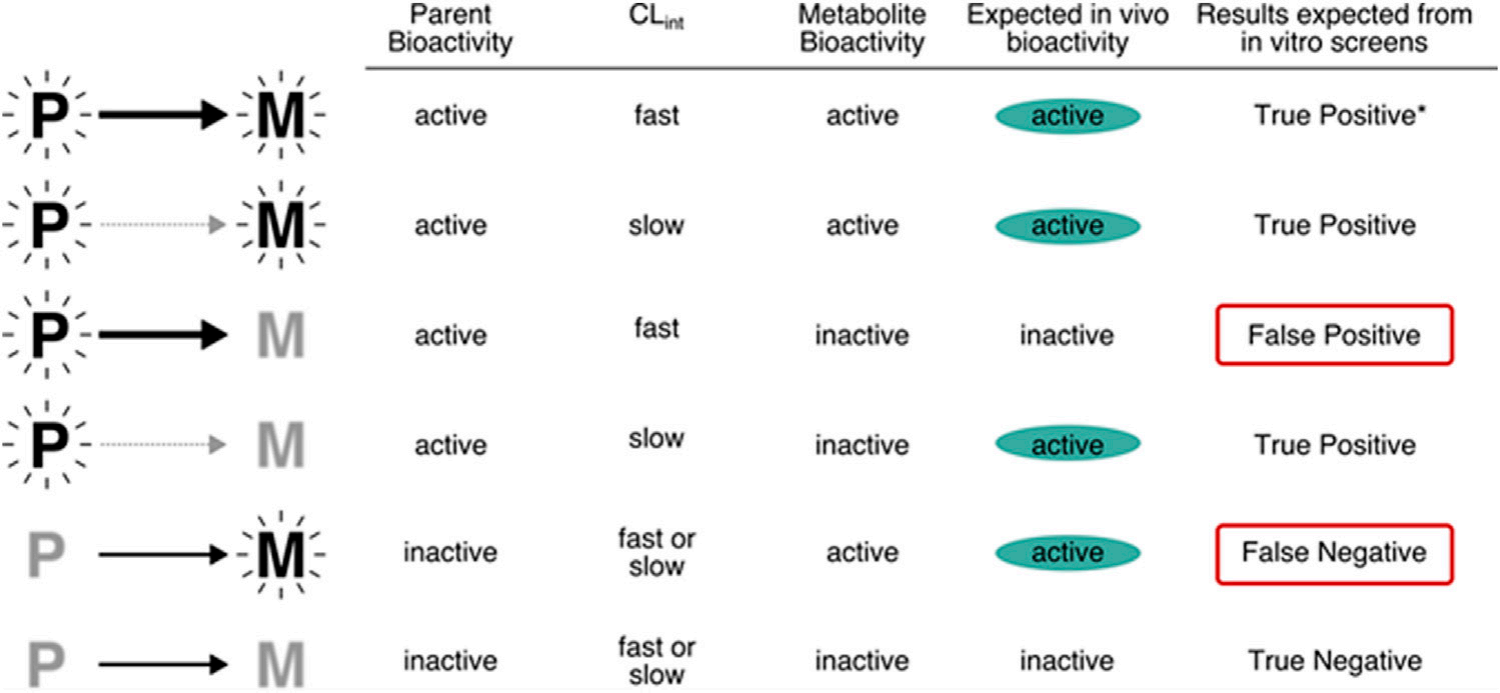
Relevance of *in vitro* assays that lack metabolism to the potential bioactivity of chemicals. Note that the top row (denoted *), will return a true positive but for the wrong reason (because the metabolite is the entity of concern *in vivo*).

It is important to make risk-based decisions based on the exposure to the active form of the chemical, whether the decisions relate to prioritization or some higher tier risk assessment. In practice, it is not a simple process, because both the tools for assessing *in vitro* bioactivity assays and for measuring *in vitro* metabolism assays may lack the full complement of enzymatic pathways required to activate a test compound and would likely misrepresent the *in vivo* phenotype. As described in Figure 5, when both parent and metabolites are active, the results expected from *in vitro* assays would be true positives. However, when the parent compound is active and the metabolite inactive, the relevance of the results would depend on the similarity of clearance *in vitro* and *in vivo.* If the compound is cleared quickly *in vivo*, spurious effects by the parent might be observed in a cell culture system that lacks the appropriate metabolic capability. In contrast, when the parent compound is inactive and the metabolite is active, the results from *in vitro* screens that lack the appropriate metabolic capability could be false negatives. For example, the phthalate esters (diethylhexyl phthalate, di-n-butyl phthalate) are antiandrogenic *in vivo* due to hydrolysis to the monoester metabolites. *In vitro* , the monoester metabolites inhibit testosterone, but parent chemicals do not (Balbuena et al., 2013; https://comptox.epa.gov/dashboard/chemical/invitrodb/DTXSID2025680). In this case, a false negative could be produced if the active metabolites were not identified by QSAR of *in vitro* metabolism studies that included esterase activity. Current *in vitro* bioactivity assays rarely consider metabolism, and when they do, the assay conditions are unlikely to mimic *in vivo* metabolite profiles. For example, the most common approach to including metabolism is the addition of S9 fractions from rat hepatocytes, often following induction of specific Phase I pathways using chemicals such as Arochlor 1254 or phenobarbital (Elliott et al., 1992; Ooka et al., 2020.). Recombinant expressed enzymes can also be applied to obtain information on metabolism, including the contribution of a single metabolic enzyme or a combination of isozymes to the biotransformation of the chemical, and the identification of potential metabolites (Hariparsad et al., 2006). The results from the expressed system can also support screening for toxicity of the metabolites (Hariparsad et al., 2006). From the QIVIVE point of view, another advantage of recombinant systems is the capability of providing human variability information when combined with enzyme abundance data (Lipscomb et al., 2003; Punt et al., 2010).

While identifying every single metabolite is likely not feasible for any given test compound or decision context, some understanding of the potential for production of a reactive metabolite and the rate of clearance of major metabolites that raise structural alerts for toxicity is crucial for any NAM-based risk assessment strategy. Hence, some combination of *in silico* tools and *in vitro* assays will be essential to inform the number of potential toxic metabolites, especially at early stages that are intended to facilitate prioritization.

At this time, no single method for metabolite prediction would be sufficient for assessing metabolite formation. Coupling predictive tools with read-across from literature reviews should aid in identifying the mostly likely metabolites to be formed. As a result, metabolites found to be common across all three sources of information—literature review, metabolite prediction software and direct *in vitro* identification - would be the most likely candidates for follow-up testing. To identify if metabolism plays a major role in response, the most frequently identified metabolites could be tested. For example, compounds with characterized activity in HT screening efforts (*e.g.*, Tox21, ToxCast), having structural flags for genotoxicity, or that are metabolites expected to be formed might be prioritized for testing. Criteria for inclusion of predicted metabolites for further testing would certainly vary according the decision context. Additionally, if a metabolite had structural characteristics that indicate that it could be more persistent or bioaccumulative than the parent compound, this could also signal the need for follow-up testing.

However, collection of literature data would likely prove to be the best option only for those few compounds that that have been extensively studied. An alternative to looking through various literature data for poorly characterized compounds would be to develop a more efficient experimental strategy for metabolite identification. A rapid metabolite identification approach based on computer-aided spectrum analysis has the potential to significantly increase throughput and confidence in incorporating metabolites in risk-based decision makings (Zamora et al., 2013).

## Conclusion

Significant progress has been made internationally in redefining the paradigm of toxicology testing to support reducing the use of animals and enhancing human risk assessment through the implementation of more human relevant *in vitro*-only strategies. Considerable progress has also been made in developing and applying screening approaches that integrate HT methods with tiered-testing strategies to support a weight-of-evidence approach to chemical safety decision making. HT-IVIVE is an important tool that utilizes *in vitro* experimental data to predict equivalent dose levels *in vivo* via dosimetry. However, there remain significant gaps in both *in vitro* and *in silico* tools related to metabolism that need to be addressed to improve the efficiency and confidence in HT-IVIVE. To address these gaps, the *in vitro* metabolism field requires significant improvements in the rapid identification of likely metabolites, characterization of metabolite formation and clearance processes, and estimation of metabolite kinetics. This task will require a collaborative effort across *in silico*, *in vitro* and analytical chemistry platforms.
